# Current Status of Canine Umbilical Cord Blood-Derived Mesenchymal Stem Cells in Veterinary Medicine

**DOI:** 10.1155/2018/8329174

**Published:** 2018-07-15

**Authors:** Tania Sultana, Soojung Lee, Hun-Young Yoon, Jeong Ik Lee

**Affiliations:** ^1^Regenerative Medicine Laboratory, Center for Stem Cell Research, Department of Biomedical Science and Technology, Institute of Biomedical Science and Technology, Konkuk University, Seoul, Republic of Korea; ^2^Regeniks Co. Ltd., Seoul, Republic of Korea; ^3^Department of Veterinary Surgery, College of Veterinary Medicine, Konkuk University, Seoul, Republic of Korea; ^4^Department of Veterinary Obstetrics and Theriogenology, College of Veterinary Medicine, Konkuk University, Seoul, Republic of Korea

## Abstract

Stem cell therapy has prompted the expansion of veterinary medicine both experimentally and clinically, with the potential to contribute to contemporary treatment strategies for various diseases and conditions for which limited or no therapeutic options are presently available. Although the application of various types of stem cells, such as bone marrow-derived mesenchymal stem cells (BM-MSCs), adipose tissue-derived mesenchymal stem cells (AT-MSCs), and umbilical cord blood-derived mesenchymal stem cells (UCB-MSCs), has promising potential to improve the health of different species, it is crucial that the benefits and drawbacks are completely evaluated before use. Umbilical cord blood (UCB) is a rich source of stem cells; nonetheless, isolation of mesenchymal stem cells (MSCs) from UCB presents technical challenges. Although MSCs have been isolated from UCB of diverse species such as human, equine, sheep, goat, and canine, there are inherent limitations of using UCB from these species for the expansion of MSCs. In this review, we investigated canine UCB (cUCB) and compared it with UCB from other species by reviewing recent articles published from February 2003 to June 2017 to gain an understanding of the limitations of cUCB in the acquisition of MSCs and to determine other suitable sources for the isolation of MSCs from canine. Our review indicates that cUCB is not an ideal source of MSCs because of insufficient volume and ethical issues. However, canine reproductive organs discarded during neutering may help broaden our understanding of effective isolation of MSCs. We recommend exploring canine reproductive and adipose tissue rather than UCB to fulfill the current need in veterinary medicine for the well-designed and ethically approved source of MSCs.

## 1. Introduction

In the last 20 years, stem cells have received ample attention from researchers in both human and veterinary medicine for their functional characteristics and therapeutic potential in different applications [1–4]. The number of animals previously treated in veterinary medicine provides a consequential basis for estimating the effectiveness of stem cell therapy in the treatment of different diseases [5, 6]. Nearly all types of animal tissues can be repaired or regenerated by the explicit action of stem cells [7], which exhibit high potential for propagation and differentiation [8]. Moreover, animal models are extensively used to examine the properties and promising potential of stem cells for reasonable application in human medicine in the future. Consequently, human and veterinary medicine are intertwined in the emerging field of stem cell research. Pioneering innovations in stem cell research have been accomplished by the collaboration of clinical and veterinary scientists.

For instance, adult stem cells isolated from various sources, mainly bone marrow- (BM-) and adipose tissue- (AT-) derived stem cells, have been widely used for the treatment of different animal diseases [9, 10]. As in human medicine, adult mesenchymal stem cells (MSCs) play an important role in veterinary medicine for the treatment of acute injury and chronic disorders. In brief, MSCs, also known as marrow stromal cells [11] or mesenchymal progenitor cells, are considered the most heavily utilized stem cells in the field of regenerative medicine and tissue engineering [12, 13] to overcome the complications and limitations of gene-based therapies. Currently, MSCs are used in clinical cell therapies and trials in many countries [14] for their *in vitro* expansion, notable multilineage differentiation potential [15, 16], capability to treat tissue injury [17, 18], viability after long-term storage by cryopreservation [19], support of hematopoietic stem cell (HSC) expansion as feeder cells [20], and immunomodulatory properties [21, 22]. These extensively applied cells were first depicted by Friedenstein et al. as a cell population analogous to fibroblasts [23]. They have the potential to differentiate into numerous cell types such as osteoblasts, adipocytes, cardiomyocytes, chondrocytes, hepatocytes, and brain cells [24–35]. These cells can be isolated from BM, AT, peripheral blood, skeletal muscle, connective tissue of the dermis, and Wharton's jelly (WJ) as well as umbilical cord blood (UCB) [30, 36–39].

Although BM represents an abundant source of MSCs [33, 40] in the field of tissue engineering and cell-based therapy, harvesting of cells is invasive with a stringent donor age requirement and increased donor site morbidity [41–46]. Therefore, UCB has been identified as an ideal alternative source in terms of ease of accessibility as well as reduced morbidity. UCB carries a large number of MSCs per volume, which are more flexible and pluripotent than bone marrow-derived mesenchymal stem cells (BM-MSCs) [38, 47]. Additionally, it has been proposed that umbilical cord blood-derived mesenchymal stem cells (UCB-MSCs) are not as mature as other stem cells and may not induce alloreactive responses that harmonize the immune system [32, 48, 49] and have the lower carcinogenic potential [50].

Nevertheless, although the presence of HSCs and their isolation from UCB are well established [51–54], the statistics concerning the existence of UCB-MSCs are contentious and require further evaluation. Earlier experiments to isolate UCB-MSCs from different species have either been aborted, have been time-consuming and onerous [55–57], or have been only 30–60% effective under suitable conditions [38, 39, 58–63].

UCB-MSCs can be isolated from different species such as human [37, 58, 59, 64–74], sheep [75, 76], equine [77–79], canine [80–84], and goat [85]. Human UCB-MSCs (hUCB-MSCs) are collected noninvasively at the time of delivery, whereas sheep UCB is collected intrusively by the surgical intrauterine approach [75, 76]. Equine UCB represents a noninvasively and nontraumatically retrieved source of stem cells, with potentially excellent cellular characteristics including proliferation capacity, immune tolerance, and differentiation potency [78]. It is reported that UCB from more than 100 mares and foals has been collected safely and efficaciously. The enrichment rate of MSCs from equine UCB is very high compared with that of other species [86].

Canine and feline are mainly considered companion animals rather than laboratory animals. Generally, pet owners prefer their pet (canine and feline) to be neutered (spayed or castrated) for the purpose of sterilization. After spay, canine UCB (cUCB) is impossible to collect, as the females lose their reproductive capability. In this view, cUCB raises the problem of limited availability. Yet, there are a few studies on the successful isolation and application of canine UCB-MSCs (cUCB-MSCs).

Even though UCB-MSCs are the latest tool and have already been commercialized for human medicine, the progress of UCB-MSC research in veterinary medicine is at a standstill. Therefore, veterinary medicine must overcome the major challenges of UCB-MSCs. We know that cUCB-MSCs have a great impact on veterinary medicine. Thus, cUCB-MSCs should be further explored to boost the supply thereof. In this respect, this is the first review that highlights the limitations of cUCB for the isolation of MSCs and suggests another significant source of MSCs in canine.

## 2. Umbilical Cord Blood and Mesenchymal Stem Cells

The umbilical cord, also known as the navel-string or birth cord, is the channel between the growing placenta and fetus. In the course of prenatal development, the umbilical cord is a physiological and inherent part of the fetus. Typically, the umbilical cord consists of two arteries (the umbilical arteries) as well as one vein (the umbilical vein) buried in WJ, a gelatinous element composed largely of mucopolysaccharides. The two umbilical arteries transfer deoxygenated, nutrient-diminished blood away, whereas the umbilical vein carries oxygenated, nutrient-boosted blood to the fetus. The umbilical cord is covered by an epithelium obtained from the enveloping amnion.

UCB has been utilized as a rich and readily available alternative source of primitive and unspecialized stem cells since 1988 [87]. The blood remaining in the umbilical vein after birth is an abundant source of hematopoietic stem and progenitor cells. This criterion makes UCB an allogenic donor source that can be applied in a variety of hematologic, pediatric, genetic, oncologic, and immunologic disorders [53, 88–90]. Fresh UCB is also an auspicious source of non-HSCs such as MSCs, endothelial cells, and unrestricted somatic stem cells [30, 91–93]. Although several previously published articles reported the identification of MSCs in UCB [38, 39], some authors debated the existence of MSCs in UCB and declared that only HSCs are present therein [56, 94]. The origin of MSCs in UCB is unrecognized, but it is possible that the cells are discharged from the fetal liver or bone marrow into the fetal circulation [65]. Presently, the isolation of MSCs from UCB has a lower success rate compared with BM-MSCs (63% versus 100%) [67]. AT is another alternative source that can be accessed less invasively and repeatedly with an easy procedure, resulting in larger quantities of fresh MSCs [95].

## 3. Process of Article Selection

We utilized the search engines PubMed and Google Scholar to gather a list of publications and manuscripts detailing research, employment, or isolation of MSCs from human and animal subjects from February 2003 to June 2017. For a report to be included in this survey, it must have contained “Umbilical Cord Blood-derived Mesenchymal Stem Cells (UCB-MSCs)” in either the heading or the abstract. The keyword “Umbilical Cord Blood-derived Mesenchymal Stem Cells (UCB-MSCs)” combined with “human,” “equine,” “sheep,” “goat,” and “canine” was used to generate the list. We did not include any review articles in our survey. Highly relevant articles were initially determined by the heading and abstract, followed by a further examination to confirm whether the collection of UCB was from human or animal subjects. We accept that this search technique was not encyclopedic, as there are many more articles in journals that are not included in PubMed or Google Scholar. We evaluated the listed studies by different characteristics such as the weight of the species, final blood volume, and enrichment rate of MSCs.

For studies in which UCB-MSCs were isolated from humans, the key areas recognized were ethics (informed consent was obtained), route of delivery, UCB unit and volume, and MSC success rate. For studies in which UCB-MSCs were isolated from animals, the key areas noted were ethics (reporting of omission and approval of the study by the Animal Care and Use Committee), study design (distribution of groups and sample number and volume), route of delivery, and experimental animals (species, breed, and weight).

## 4. UCB-MSC Articles Entailing Human and Animal Sources

The electronic search singled out 130 articles. A total of 20 articles based on human and animal sources of UCB-MSCs were considered based on the heading, abstract, and content (species, UCB unit and volume, and MSC success rate). Review articles, duplicates, and irrelevant papers were removed. In total, 55% (11/20) of articles reported human subjects, and 45% (9/20) of papers reported animal subjects, such as equine, sheep, goat, and canine (Table 1). Nine articles regarding human subjects stated that UCB was collected with the informed consent of the mothers, while four studies detailing animal subjects received approval from an animal ethics committee (Table 2).

## 5. Success Rate of MSC Isolation from UCB

Some researchers successfully isolated 33.3%–60% of hUCB-MSCs under different cell culture conditions [69, 71]. Bieback et al. reported that a net volume of more than 33 mL of UCB and a mononucleated cell (MNC) count higher than 1 × 10^8^ are difficult to achieve for the isolation of UCB-MSCs from human subjects. However, they were capable of increasing the success rate from 29% to 63% [37]. Jin et al. reported the isolation of 50% of UCB-MSCs from 24 pregnant women [67]. When the blood volume exceeds 90 mL, 90% of MSCs from UCB can be isolated [66, 70]. Liu et al. stated that the efficacy of MSC isolation from UCB can reach 75% (*n* = 144) [72]. Sibov et al. and Johannes et al. demonstrated that more than 70 mL of UCB is required for the successful isolation of MSCs [70, 73]. On the other hand, Thomas et al. isolated equine UCB-MSCs from a volume of 42 mL at a 100% success rate with PrepaCyte-EQ (PEQ) medium. The same authors isolated MSCs with a success rate of 57% from a volume of 65–250 mL of equine UCB [77, 78]. Different authors have investigated the isolation of MSCs from UCB of goat and sheep (Table 3) [76, 85].

Although Kang et al. and Jang et al. separately continued their experiments with cUCB-MSCs, they did not explicitly provide any information about the blood volume and number of canine subjects they used [80, 83]. Lim et al. successfully isolated MSCs from an exceedingly low UCB volume of 8 mL [84]. As the UCB volume of canine is insufficient to isolate MSCs, another author utilized the blood of canine fetus heart along with UCB [82] (Tables 4 and 5).

## 6. MSCs in Human and Veterinary Medicine

The application of stem cells in cell-based therapies and tissue engineering is increasing to overcome the complications and hurdles of gene therapy. In both animals and human, stem cell implantation can serve as an advanced treatment for some incurable conditions such as bone fracture malignancies [96, 97], spinal cord injuries [98], and genetic disorders [99]. Tables 6 and 7 summarize the application of MSCs in the treatment of a wide range of diseases in preclinical studies of experimental animal models and veterinary clinical studies of animals with naturally occurring diseases. In human medicine, MSC products have already been developed and approved in South Korea. Cartistem® (Medipost), the world's first allogenic hUCB-MSC drug, has gained popularity in South Korea since January 2012 for the treatment of cartilage injury and osteoarthritis, and efforts are presently being made to broaden its indications for different target diseases. However, the isolation and characterization of stem cells obtained from numerous tissues and sources have led to highly detracting arguments regarding stem cell therapy [100–102].

Stem cell researchers worldwide are endeavoring to acquire the ideal or optimal autologous or allogenic MSCs, not only from human but also from equine, canine, and feline, to treat diseases such as musculoskeletal diseases, degenerative arthritis, atopic dermatitis, myocardial disease, chronic renal failure, and nerve damage [103]. Based on the concept of “One Health,” new synergistic therapies such as stem cell therapies for human and veterinary medicine are in increasing demand and developed in collaboration—the time for focus on human and animal health is now [104].

## 7. Therapeutic Potential and Sources of MSCs

MSCs, specifically multipotent stem cells, have the capability to generate adipogenic, chondrogenic, osteogenic, and myogenic as well as endothelial cell lineages [14, 37]. Because of these possibilities, there has been an increased focus on the isolation of MSCs from different sources for transplantation and tissue engineering. MSCs have been isolated from diverse adult-derived sources such as BM, AT, lung, heart, peripheral blood, synovium skeletal muscle, dermis, and dental pulp as well as from fetal or neonatal tissues such as amniotic fluid and membrane, placenta, UCB, cord vein, WJ, and umbilical cord [59, 105–108]. BM is a bountiful source of MSCs; however, AT is a reliable source of MSCs with the best frequency, and UCB seems to be a remarkable alternative source that allows expansion for a greater number of MSCs [59, 64, 67].

## 8. UCB-MSCs from Human Sources

The frequency of MSCs in hUCB is a point of controversy among researchers [56, 57, 107]. This is attributed to the persistent difficulties in the isolation of UCB-MSCs. Most researchers have adopted suitable conditions from the literature to enrich the recovery of UCB-MSCs, including choosing full-term UCB units, storing for no more than 15 h, and obtaining an MNC count above 1 × 10^8^ as the selection criterion for UCB units [37]. While Rebelatto et al. declared that sample volume did not correlate with MSC isolation [62], others have suggested that sample volume (minimum 33 mL [37] or 45 mL [109]) is a critical parameter. The enrichment rate of MSCs has varied broadly from 10% to 60% [110–112]. Namely, of 644 UCB units were subjected to MSC isolation in diverse studies, only 167 successful outgrowths have been recorded (26% success rate) [38, 58–60, 62, 113]. A high enrichment rate of 90% MSCs was obtained when the UCB volume was more than 80 mL [66, 70].

Many groups have reported an isolation efficiency of 65% using numerous culture methods such as reduction of monocytes and lymphocytes from MNCs before cell seeding, development of cells under hypoxia, and the inclusion of cytokines, platelet lysate, or medium supplements [64, 66, 114, 115]. Other published studies accomplished isolation of MSCs from human UCB with up to 40–90% efficiency by adopting different parameters [20, 37, 65–67, 69, 72].  Table 8 represents the yield rate of UCB-MSCs isolated from human and animal sources fulfilling special criteria.

## 9. UCB-MSCs from Animal Sources

**Figure 1:**
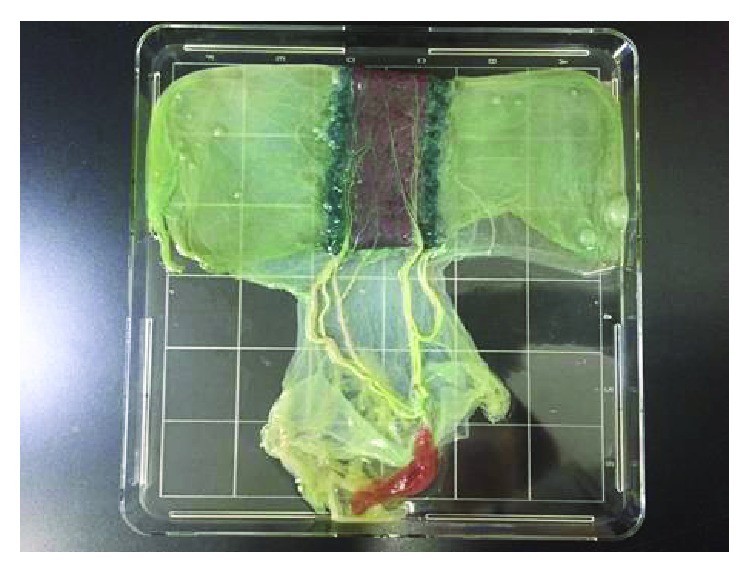
The canine umbilical cord with the placenta and amniotic sac.

Successful isolation of MSCs (up to 100%) from equine UCB has been reported with the use of special culture media with a large volume of 42–250 mL [77, 78]. Although the isolation of MSCs from sheep and goat was successful, the researchers did not reveal any specific parameters that resulted in an effective number of MSCs [75, 76, 85]. Based on our literature survey, we have demonstrated that a large volume of UCB with some other special determinants is a principal factor to achieve high MSC yield. As the blood volume of cUCB is too low to yield an efficient number of MSCs, cUCB may be recognized as an unsuitable source of MSCs. Lim et al. worked with a 58 kg mongrel dog, which very rarely have more than 8 mL of UCB [84]. In two independent studies by Kang et al. and Jang et al., UCB from multiple dogs was collected to obtain the desired number of MSCs [80, 83]. As the amount of UCB from canine cannot produce a sufficient number of MSCs, another researcher collected blood from the canine fetal heart and successfully obtained MSCs [82], which creates an inconsistency and ethical problems in the pursuit of animal experiments. Our attempt to isolate MSCs from the umbilical cord (Figure 1) [116] of three healthy dogs resulted in an average sample volume of 1 mL and a yield rate of 66% (unpublished data).

## 10. UCB from Companion Animals

Nowadays, canine cesarean sections (C-sections) are decreasing because of the increase in spaying or castration to prevent the birth of unwanted litters, extend longevity and promote pet health, and possibly mitigate undesirable behaviors. More precisely, spaying can help prevent uterine infections and breast tumors. Concurrently, castration of male companion animals precludes testicular cancer as well as some prostate problems. The traditional time for neutering is before the onset of sexual maturity, as long as the animal is healthy. Spaying involves abdominal surgery to remove the ovaries and uterus, whereas castration involves elimination of the testes, resulting in infertility. Under these circumstances, while acquiring cUCB is normally challenging, these removed reproductive organs are frequently accessible in veterinary clinics. Specifically, the ovaries and testes are often discarded as medical waste but are proven to be potent and reliable sources of MSCs. MSCs from these reproductive organs (testis and ovary with adjacent portions) with high differentiation potentiality have already gained popularity.

These MSCs are able to differentiate into three mesodermal cell types, including adipogenic, osteogenic, and chondrogenic, indicating multipotent properties resembling those of AT-MSCs [117]. We can thoroughly examine the presence of MSCs in canine reproductive organs and surrounding tissues (fat and connective tissue) discarded during neutering operations; these MSCs also exhibit growth characteristics indistinguishable from those of AT-MSCs. Therefore, further investigation of canine reproductive organs and tissues with the assistance of new preservation technology [118] will improve our understanding of reproductive stem cell biology to potentially augment cell-based therapies and regenerative medicine.

Unlike in humans, it is almost impossible to obtain UCB from canine immediately after natural birth, as the dam's instinct prompts her to ingest the puppy's placenta and umbilical cord. From this perspective, we have made many efforts (unpublished data) to collect cUCB from Korean veterinary clinics. However, this was difficult, as many pet owners tend to prevent their pet from breeding. Conversely, if the canine pet is pregnant, the owner generally prefers natural birth unless a medical reason necessitates C-section. Therefore, the reduction in canine C-sections in veterinary hospitals, the introduction of specialized breeders, and changes in the supply of canine make it almost impossible to obtain UCB from canine. Conducting a surgical procedure or C-section in canine solely to obtain UCB for laboratory purposes gives rise to the question of legitimacy. To solve this problem, we can use MSCs from canine AT [95] along with reproductive tissue. It has been demonstrated that MSCs from AT have higher frequency and potentiality compared with UCB-MSCs [59, 67].

## 11. Conclusions

One challenge in cellular therapy and regenerative medicine is the availability of alternative stem cell sources. Although cUCB-MSCs can proliferate and differentiate as stem cells, cUCB is not an ideal source because of low volume and ethical considerations of using a companion animal. In this regard, we suggest broadening the investigation of canine reproductive tissue and AT, instead of UCB, as the preferred source of MSCs.

## Figures and Tables

**Table 1:** Articles included in this survey regarding UCB-MSCs from different species.

Sources of UCB	Reference	Number of articles
Human	[20, 37, 58, 65–67, 69–73]	11
Animal	[75–78, 80, 82–85]	9

**Table 2:** Summary of ethical approval in studies that reported the collection of UCB from human and animal subjects.

Ethics	Reference	
	Yes	No
Informed consent from UCB donors	[20, 37, 58, 65–67, 69, 70, 73]	[71, 72]
Approval from animal ethics committee	[75, 80, 84, 85]	[76–78, 82, 83]

**Table 3:** Summary of papers that reported data regarding the isolation of UCB-MSCs from human, equine, sheep, and goat.

Species	Breed	Route of delivery	UCB unit	UCB volume (mL)	Frequency of MSCs (%)	Reference
Human	—	Vaginal	10	50	60	[71]
Human	—	Cesarean	35	—	40	[69]
Human	—	Vaginal	13	60	46	[20]
Human	—	Vaginal	59	108	63	[37]
Human	—	Vaginal	24	—	50	[67]
Human	—	Vaginal	—	≥80	90	[66, 70]
Human		Cesarean	456	≥90	90.9	[65]
Human	—	Vaginal	144	—	75	[72]
Human	—	Vaginal	9205	40–321	—	[73]
Human	—	Vaginal	30	—	35	[58]
Equine	—	Vaginal	7	65–250	57	[78]
Equine	—	Vaginal	5	42	100	[77]
Goat	Mongrel	Vaginal	—	—	—	[85]
Sheep	Santa Ines	Surgical procedure	5	100	—	[76]
Sheep	—	Surgical intrauterine approach	4	10	—	[75]

**Table 4:** Summary of papers that reported data regarding the isolation of MSCs from cUCB.

Source of MSCs	Breed	Weight (kg)	Route of delivery	UCB Unit	UCB Volume (mL)	Reference
cUCB	Mongrel	58	C-section	1	8	[84]
cUCB and blood of canine fetus heart	—	—	—	—	—	[82]
cUCB	—	—	C-section	—	—	[83]
cUCB	Beagle	10.1	C-section	7	—	[80]

C-section: Cesarean section.

**Table 5:** Summary of purposes of cUCB studies.

Purpose of experiment	Reference
Transplantation of cUCB-MSCs in spinal cord injured dogs	[84]
Isolation and characterization of cUCB-MSCs	[82]
Implantation of cUCB-MSCs mixed with *β*-TCP to enhance osteogenesis	[83]
Comparison of osteogenic potential of canine MSCs derived from AT, BM, UCB, and WJ	[80]

*β*-TCP: beta-tricalcium phosphate.

**Table 6:** Preclinical studies with MSCs.

Cell source	Cell type	Pathology	Outcome	Reference
Rat	Human AD-MSC	Peripheral nerve injury	Repair of nerve	[119]
Rat	Human AD-MSC	Cerebral ischemia	Repair of nerve	[120]
Mouse	Human UCB-MSC	Hind limb ischemia	Repair of artery	[121]
Rat	Human UCB-MSC	Cavernosal nerve injury	Improved function	[122]
Rat	Allogenic AD-MSC	Peripheral nerve injury	Repair of nerve	[123]
Rat	Allogenic BM-MSC	Skin wound	Repair of skin	[124]
Rat	Autologous BM-MSC	Spinal cord injury	Repair of nerve	[125]
Rat	Autologous AD-MSC	Spinal cord injury	Repair of nerve	[126]
Rat	Mouse SC-MSC	Erectile dysfunction	Improved function	[127]
Rabbit	Human AD-MSC	Spinal cord injury	Repair of nerve	[128]
Rabbit	Human UCWJ-MSC	Normal	No immune rejection	[129]
Rabbit	Porcine S-MSC	Osteochondral defect	Immune rejection	[130]
Rabbit	Allogenic S-MSC	Articular cartilage defect	Repair of defect	[131]
Rabbit	Allogenic BM-MSC	Articular cartilage defect	Repair of defect	[132]
Rabbit	Allogenic/autologous BM-MSC	Bone defect	Repair of defect	[133]
Porcine	Autologous BM-MSC	Articular cartilage defect	Repair of defect	[134]
Porcine	Mouse BM-CMSC	Vocal fold wound	Repair of vocal fold	[135]
Porcine	Allogenic S-MSC	Articular cartilage defect	Repair of defect	[136]
Canine	Allogenic BM-MSC	Bone defect	Repair of defect	[137]
Canine	Allogenic BM-MSC	Cardiac ischemia	Improved function	[138]
Canine	Allogenic BM-MSC	Myocardial infarction	Improved function	[139]
Canine	Allogenic BM-MSC	Skin wound	Repair of skin	[140]
Canine	Allogenic BM-MSC	Normal	Inflammation, tubular necrosis, mineralization, and fibrosis of kidney	[141]
Canine	Allogenic AD-MSC	Spinal cord injury	Repair of nerve	[80]
Canine	Allogenic UCB-MSC	Spinal cord injury	Repair of nerve	[84]
Canine	Autologous BM-MSC	Disc degeneration	Repair of nerve	[142, 143]
Canine	Autologous BM-MSC	Bone defect	Repair of defect	[144]
Canine	Autologous BM-MSC	Static nerve injury	Repair of nerve	[145]
Canine	Autologous BM-MSC	Osteochondral defect	Repair of defect	[146]
Canine	Autologous BM-MSC	Myocardial infarction	Improved function	[147]
Canine	Autologous BM-MSC	Osteonecrosis of the femoral head	Repair of blood vessel	[148]
Canine	Autologous BM-MSC	Oral ulcer	Repair of ulcer	[149]
Canine	Autologous BM-MSC	Diabetes	Improved function	[150]
Canine	Autologous BM-MSC	Vocal fold wound	Repair of vocal fold	[151, 152]
Canine	Autologous BM-MSC	Periodontal defect	Repair of defect	[153]
Canine	Autologous BM-MSC	Periodontal class II furcation defect	Repair of defect	[154]
Canine	Allogenic/autologous BM-MSC	Spinal cord injury	Repair of nerve	[155]
Canine	Autologous AD-MSC	Spinal cord injury	Repair of nerve	[156]
Canine	Autologous MSC	Chondral defect	Repair of defect	[157]
Canine	Allogenic AD-MSC	Thoracolumbar intervertebral disc disease	Improved clinical sign	[158]
Equine	Allogenic AD-MSC	Superficial digital flexor tendonitis	Repair of tendonitis	[159]
Equine	Autologous BM-MSC	Superficial digital flexor tendonitis	Repair of tendonitis	[160]
Equine	Autologous BM-MSC	Osteoarthritis	Repair of osteoarthritis	[161]
Equine	Allogenic/autologous BM-MSC	Normal	Enhancement of MSC	[162]
Dolphin	Autologous AD-MSC	Normal	Cell collection success	[163]
Caprine	Autologous BM-MSC	Osteoarthritis	Repair of osteoarthritis	[164]

AT-MSC: adipose tissue-derived mesenchymal stem cells; UCB-MSC: umbilical cord blood-derived mesenchymal stem cells; BM-MSC: bone marrow-derived mesenchymal stem cells; SC-MSC: skeletal muscle-derived mesenchymal stem cells; S-MSC: synovium-derived mesenchymal stem cells; UCWJ-MSC: umbilical cord Wharton's jelly-derived mesenchymal stem cells.

**Table 7:** Veterinary clinical studies with MSCs.

Cell source	Cell type	Pathology	Outcome	Reference
Canine	Autologous BM-MSC	Gastrocnemius tendon injury	Repair of tendon	[165]
Canine	Autologous BM-MSC	Chronic Chagas cardiomyopathy	Improved function	[166]
Canine	Autologous AD-MSC	Chronic anconitis	Repair of anconitis	[95]
Canine	Autologous AD-MSC	Arthritis, patella luxation	Repair of arthritis	[167]
Canine	Autologous AD-MSC	Chronic arthritis of hip joint	Repair of arthritis	[168]
Canine	Autologous AD-MSC	Atopic dermatitis	Repair of dermatitis	[169]
Canine	Allogenic AD-MSC	Lumber herniated intervertebral disc	Repair of nerve	[170]
Canine	Allogenic AD-MSC	Hip dysplasia	Improved function	[9]
Canine	Autologous AD-MSC	Chronic osteoarthritis related to hip dysplasia	Reduced lameness	[171]
Canine	Autologous AD-MSC	Severe osteoarthritis	Reduced lameness	[172]
Canine	Human ESC-MSC	Anal furunculosis	Recovery of fistulas	[173]
Feline	Autologous AD-MSC	Chronic kidney disease	Moderate improvement in GFR	[174]
Feline	Allogenic AD-MSC	Chronic kidney disease	Modest improvement in renal function	[175]
Feline	Allogenic AD-MSC	Chronic kidney disease	No adverse effect	[176]
Feline	Autologous AD-MSC	Chronic gingivostomatitis	Reduction in the severity of clinical disease	[177]
Equine	Allogenic UCB-MSC	Chronic laminitis	Repair of laminitis	[178]
Equine	Autologous BM-MSC	Tendon injury	Repair of tendon	[179]
Equine	Autologous BM-MSC	Superficial digital flexor tendon	Repair of tendon	[180]
Equine	Autologous BM-MSC	Tendinitis, desmitis	Repair of inflammation	[181]
Equine	Autologous AD-MSC	Bone spavin	Improvement in inflammatory reaction and clinical conditions	[182]
Equine	Allogenic AD-MSC	Endometriosis	Positive remodeling of endometrial tissue	[183]
Equine	Allogenic AD-MSC and PRP	Superficial digital flexor tendon	Repair of tendonitis	[184]
Equine	Allogenic AD-MSC and PRP	Tendonitis	Improved function	[185]
Dolphin	Autologous AD/BM-MSC	Skin wound	Repair of skin	[186]

AT-MSC: adipose tissue-derived mesenchymal stem cells; UCB-MSC: umbilical cord blood-derived mesenchymal stem cells; BM-MSC: bone marrow-derived mesenchymal stem cells; ESC-MSC: embryonic stem cell-derived mesenchymal stem cells; GFR: glomerular filtration rate; PRP: platelet-rich plasma.

**Table 8:** Yield rate of UCB-MSC isolation from human and equine fulfilling special criteria.

Source of UCB	Parameters		Yield rate (%)	Reference
Human	Explant method		40	[69]
Human	Large BV with HPL medium		46	[20]
Human	Large number of UCB units		50	[67]
Human	Large BV with MesenCult Proliferation kit		60	[71]
Human	ST ≤ 15 h, BV ≥ 33 mL, ≥10^8^ MNC count		63	[37]
Human	Large number of UCB units with serum-free culture medium		75	[72]
Human	Factorial experiment with large BV		≥90	[66]
Human	ST ≤ 2 h, V ≥ 90		90.9	[65]
Human	DP ≤ 37 weeks, BV ≥ 80 mL, ST ≤ 6 h		90	[70]
Equine	BV 42 mL	PEQ medium	100	[77]
		FUD medium	60	
		FD medium	20	
Equine	Large BV		57	[78]

HPL: human platelet lysate; MNC: mononucleated cell; ST: storage time; BV: blood volume; DP: duration of pregnancy; PEQ: PrepaCyte®-EQ medium; FUD: Ficoll-Paque™ PREMIUM medium loaded with undiluted whole blood, FD: Ficoll-Paque PREMIUM medium loaded with diluted whole blood.
